# FISHing Out the Hidden Enemy: Advances in Detecting and Measuring Latent HIV-Infected Cells

**DOI:** 10.1128/mBio.01433-17

**Published:** 2017-09-19

**Authors:** Vinayaka R. Prasad, Ganjam V. Kalpana

**Affiliations:** a Department of Microbiology and Immunology, Albert Einstein College of Medicine, Bronx, New York, USA; b Department of Genetics, Albert Einstein College of Medicine, Bronx, New York, USA

**Keywords:** Eradication, FISH-flow, HIV-1, latency, RNA FISH

## Abstract

The indomitable aspect of HIV-1 infection is not that HIV-1 proviral DNA is integrated into host DNA but that it can also turn itself off, remaining invisible to drug or immune surveillance. Thus, the goals of eradication include ways to precisely excise HIV-1 DNA or wake up the silent HIV-1 provirus and eliminate the infected cells thus identified. Methods to identify and fish out the latently infected cells or to delineate their characteristics are being rapidly developed. In 2016, Baxter et al. (A. E. Baxter, J. Niessl, R. Fromentin, J. Richard, F. Porichis, R. Charlebois, M. Massanella, N. Brassard, N. Alsahafi, G. G. Delgado, J. P. Routy, B. D. Walker, A. Finzi, N. Chomont, and D. E. Kaufmann, Cell Host Microbe 20:368–380, 2016, https://doi.org/10.1016/j.chom.2016.07.015) and Martrus et al. (G. Martrus, A. Niehrs, R. Cornelis, A. Rechtien, W. García-Beltran, M. Lütgehetmann, C. Hoffmann, and M. Altfeld, J Virol 90:9018–9028, 2016, https://doi.org/10.1128/JVI.01448-16) reported using the fluorescence *in situ* hybridization-flow cytometry technique to identify and quantify cells expressing HIV-1 RNA and Gag protein, as well as bearing unique cell surface markers. In a recent article in *mBio*, Grau-Expósito et al. (J. Grau-Expósito, C. Serra-Peinado, L. Miguel, J. Navarro, A. Curran, J. Burgos, I. Ocaña, E. Ribera, A. Torrella, B. Planas, R. Badía, J. Castellví, V. Falcó, M. Crespo, and M. J. Buzon, mBio 8:e00876-17, 2017, https://doi.org/10.1128/mBio.00876-17) reported a similar method that they claim to be more sensitive. With these methods, researchers are one step closer to measuring latent reservoirs and eliminating critical barriers to HIV eradication.

## COMMENTARY

HIV control has reached its final phase (1), namely, its eradication in the form of successful elimination of latent viral reservoirs in infected individuals. At present, the nature, size, and distribution of the latent HIV reservoir within a given patient are unknown. Latent reservoirs exist in peripheral blood, lymph nodes, gut-associated lymphoid tissue, T lymphocytes, and the central nervous system. Many approaches to the elimination of latently infected reservoirs have been described, including (i) precise excision of integrated proviruses via CRISPR-Cas9 (2) or other precision nuclease technologies; (ii) “shock-and-kill” approaches that involve the administration of latency reversal agents (LRAs), followed by the elimination of reactivated cells (3); and (iii) induction of deep latency (4). While each of these methods has its own strengths and weaknesses, shock and kill has received the greatest attention. Several LRAs have been identified that can reactivate transcriptionally silent proviruses—acting via one of two major mechanisms—which results in active replication, producing virus particles (5). Early clinical trials based on the principle of shock and kill have failed, however, and the precise reason for this failure is unclear.

**FIG 1:**
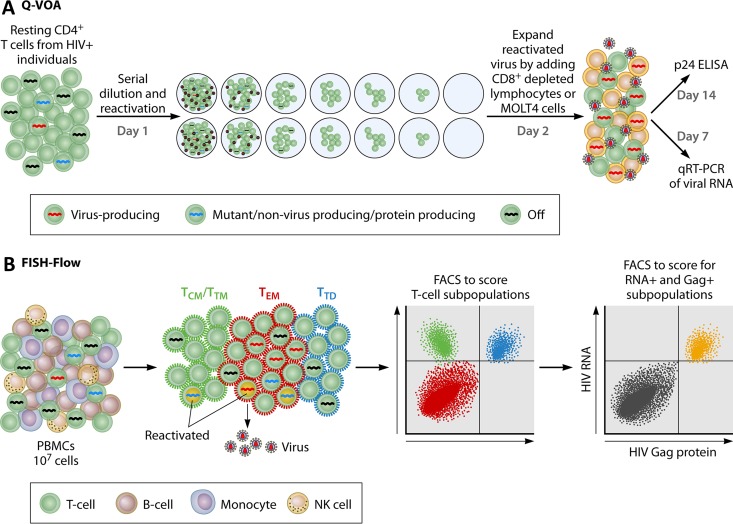
Schematic representation of the FISH-flow assay alongside that of the QVOA. (A) QVOA. The steps involve the preparation of resting CD4 T cells from HIV-infected individuals, serial dilution and reactivation (day 1), expansion of the reactivated virus (day 2), and subsequent detection of the expanded viruses by quantitation of viral RNA or protein in the supernatant by quantitative RT (qRT)-PCR or by p24 enzyme-linked immunosorbent assay (ELISA), respectively. (B) FISH-flow assay. The steps involve the use of PBMCs directly or after reactivation to score for cells expressing HIV-1 RNA and Gag protein, as well as for cell surface markers (the example shown is for T cell subset markers—shown with antibody-labeled surface markers). FACS, fluorescence-activated cell sorting.

The development of successful means of eradication has been hindered by our inability to identify latent cells with the potential to produce infectious virions and to quantitate the size of these latent reservoirs. Quantification is difficult because the number of latently infected cells is very low and they are present among a large background of cells harboring defective integrated proviral HIV DNA that are not capable of producing infectious virions. One can also measure the total amount of virus produced by cells in which HIV is actively replicating—but this will only yield the quantity of virus produced and not the quality or identity of the cells that produced it. The current gold standard assay used to measure the number of reactivated cells capable of producing infectious virions is the quantitative virus outgrowth assay (QVOA) (Fig. 1) (6), in which resting CD4 T cells isolated from an infected patient on suppressive therapy are plated in a limiting-dilution assay and activated by phytohemagglutinin and gamma-irradiated peripheral blood mononuclear cells (PBMCs), and then CD8-depleted lymphoblasts are added to expand the reactivated virus. It is expected that T cell activation will reverse the latency in these cells, resulting in the production of replication-competent HIV-1 particles. The assay typically detects 0.3 to 3 cells capable of producing virions (termed infectious units) per million cells (IUPM). Alternative methods have been described—digital droplet PCR (7), the Tat/Rev-induced limiting-dilution assay (8), both of which measure reactivated latent cells producing viral RNA, and a method to quantitatively measure viral RNA in culture supernatants upon reactivation (9)—but QVOA remains the standard because it is the only assay that quantifies the number of cells that produce infectious virus particles.

While the QVOA is a pioneering contribution to the field, it has some drawbacks. The assay is labor intensive and detects replication-competent virus as a single mixture of secreted virus with no hint of which cells the virus originated from. Furthermore, compared to a method that measures the frequency of cells with intact proviruses, QVOA underestimates the total latent virus pool (10). In addition, no qualitative determination of the nature of the virus being produced is possible. Finally, since the virus producer cells cannot be detected, no phenotyping of the producer cells is possible. A further improved version of this assay, the quantitative, qualitative virus outgrowth assay (11), does address at least one of these issues, i.e., sequencing of the viral RNA produced from the reactivated latent cells to gain insight into the nature of the reactivated virus. Overcoming the other drawbacks will be a key step toward progress in the field of HIV cure.

### Combining the power of *in situ* PCR and flow cytometry.

The report of Grau-Expósito et al. recently published in *mBio* (12), together with those of Baxter et al. (13) and Martrus et al. (14) published last year in *Cell*, *Host & Microbe*, and the *Journal of Virology*, respectively, addresses another drawback. All three employed the same cutting-edge technology, which combines the power of branched-chain amplification of cDNAs generated *in situ* within fixed T cells with that of flow cytometry, to detect HIV-1-infected cells (Fig. 1). Named the Human PrimeFlow RNA assay (EBioscience/Affymetrix), this method detects HIV-1 RNA within cells. The use of gentle fixation steps and a low temperature for hybridization with probes facilitates integration with flow cytometry, allowing the detection of both intracellular (e.g., Gag) and cell surface (e.g., CD4, CD45RO) proteins. By using this fluorescence *in situ* hybridization (FISH)-flow method, one can surface stain cells to detect phenotypic markers and intracellularly stain HIV-1 Gag protein prior to branched DNA amplification. The amplification step involves reverse transcribing HIV-1 RNA and amplifying the signal with a set of 40 to 50 individual probes targeting Gag-Pol. The final step is flow cytometry to obtain the readouts. During the many decades of HIV research, it has never been possible to track the presence of HIV RNA, Gag protein, and surface markers on the same cells. Thus, the FISH-flow method, employed in all three studies, is a powerful tool in the quest for an ideal method to measure the latent HIV-1 reservoir.

Grau-Expósito et al. (12) claim that the advantage of their assay is a higher degree of sensitivity due to both the higher number of probes used in their case and the unusual level of signal amplification achieved *in situ* (theoretical yields of up to 16,000-fold) by the branched DNA amplification used in all three studies. A major disadvantage of the RNA FISH-based assay, however, is that it does not distinguish between cells harboring functional proviruses and those harboring defective proviruses, thus leading to an overestimate of the size of the reservoir. Thus, the HIV-1-reactivated cells measured have been conservatively called HIV-1 RNA^+^ Gag^+^ cells or translation-competent HIV-infected cells (13) instead of HIV-producing cells.

The number of IUPM determined by the improved sensitivity of their method was 3.56 translation-competent cells/million cells for Baxter et al. (13) and 6 RNA- and protein-positive cells/million CD4^+^ cells for Grau-Expósito et al. (12)—both much higher than that determined by QVOA but less than that detected by reverse transcription (RT)-PCR methods. Considering that FISH-flow detects all translation-competent HIV-infected cells, this is expected. Furthermore, there was a strong correlation between the robust expression of HIV-1 RNA and Gag proteins and the downmodulation of cell surface CD4, while cells that expressed only HIV RNA did not downmodulate CD4 appreciably (12). Two of the groups also reported the downmodulation of HLA class I molecules (13, 14), and one of them showed that BST-2 is downmodulated (14), which are indirect measures of the expression of Nef/Env and Vpu, respectively. Both Grau-Expósito et al. and Baxter et al. examined the abundance of Gag^+^ and RNA^+^ cell populations in subsets of T cells. Both central and effector memory cell populations were found to contain these cell populations (12, 13). Grau-Expósito et al. further showed that effector memory cells specifically harbor latent reservoirs and that the RNA^+^ Gag^+^ cells increase upon reactivation—these findings need to be confirmed by others. The ability of FISH-flow to detect not only HIV RNA but also HIV-1 Gag protein in the cell and surface markers enables one to learn much more about the cells being reactivated from latency, and thus, it is a better tool with which to study HIV latency.

One of the major challenges for these assays is how to further distinguish subclasses of cells within the reactivated latent HIV-infected cells, because not all of the RNA^+^ Gag^+^ cells will be capable of producing infectious virions. Such cells need to be distinguished from those that are merely translation competent (RNA^+^ Gag^+^), which will allow one to precisely measure latently infected cells. Furthermore, as shown by fluorescence images in some of these reports (12, 13), it is possible to visualize bright spots of RNA within cells that are not necessarily single RNA molecules. Methods that employ the detection of single RNA molecules in reactivated cells may provide means to quantitate RNA that will help us understand reactivation kinetics.

The availability of the RNA FISH-flow method could shed light on the features of HIV-infected cells *in vivo* in a variety of clinical settings. We know that HIV infects cells expressing specific receptors (CD4 receptor and CCR5/CXCR4 coreceptor) within specific cell types (macrophages, T cells, dendritic cells, etc.), and the anatomical locations of these cells are also known. The development of the RNA FISH-flow method now makes it possible to identify the surface markers on cells that express viral RNA and Gag protein *in vivo*. Thus, one can now begin addressing the question of precisely what types of cells are infected *in vivo*, which HIV-infected cells disappear upon treatment initiation or during vaccination, and which cells are the first to reappear during treatment interruption or in other scenarios.

More importantly, this method will make it possible to determine surface markers on latent cells, providing, for the first time, a means of tracking latently infected cells or newly reactivated cells *in vivo*. One such marker, CD32a, has recently been reported by Descours et al. (15). In fact, Grau-Expósito et al. reported that when they infected unstimulated PBMCs *ex vivo*, HIV-infected T cells did upregulate the expression of the newly identified marker of latently infected cells, CD32. However, the proportion of CD32a-bearing cells within the activated cells was much smaller than that observed by Descours et al. (14). An additional impact of this method on HIV cure programs comes from its ability to measure the efficiency of reactivation by different LRAs. For example, Grau-Expósito et al. showed that PMA/ionomycin stimulation and romidepsin differed starkly in the ability to stimulate cells to reactivate HIV (12). Martrus et al. also reported that romidepsin is much slower than tumor necrosis factor alpha stimulation by employing the latency cell line J89.

Novel methods of quantitating latently HIV-infected cells, as well as improvements of existing methodologies, are being rapidly developed. Several further attempts to improve the QVOA (16, 17) complement ongoing efforts to find novel ways to detect and measure the inducible, replication-competent HIV-1 reservoir (18). These developments paint a bright future for a path toward the eradication of HIV-1, a pathogen that is so good at hiding.
